# Association between dietary fat quality indices with anthropometric measurements in children and adolescents

**DOI:** 10.1186/s12887-022-03307-0

**Published:** 2022-05-02

**Authors:** Maedeh Mozafarinia, Motahar Heidari-Beni, Behnood Abbasi, Roya Kelishadi

**Affiliations:** 1 Department of Nutrition, Electronic Health and Statistics Surveillance Research Center, Science and Research Branch, Islamic Azad University, Tehran, Iran; 2 Department of Nutrition, Child Growth and Development Research Center, Research Institute for Primordial Prevention of Non-Communicable Disease, Isfahan University of Medical Sciences, Isfahan, Iran; 3 Department of Pediatrics, Child Growth and Development Research Center, Research Institute for Primordial Prevention of Non-Communicable Disease, Isfahan University of Medical Sciences, Isfahan, Iran

**Keywords:** Dietary Fats, Lipophilic index, Thrombogenic index, Anthropometry

## Abstract

**Background:**

The association between anthropometric measures and dietary fat quality indices is unclear in pediatric age groups. The present study aimed to assess the association between dietary lipophilic index (LI) and thrombogenic index (TI) as dietary fat quality indices with anthropometric measurements in children and adolescents.

**Method:**

This nationwide cross-sectional study was conducted on 4323 students aged 6-18 years that were selected by multistage cluster sampling from 31 provinces of Iran. Dietary intake was collected using a validated food frequency questionnaire and dietary LI and TI were calculated by formula. Data on anthropometric measures were collected by standard protocols.

**Results:**

The multivariate regression analysis revealed that TI and LI had inverse association with neck circumference Z-score (β = 0.11, *p* = 0.013 and β = 0.12 *p* = 0.006, respectively). There was a positive correlation between LI with height Z-score (β = 0.12, 95% CI: 0.01, *p* = 0.009). However, there was no significant association between LI and TI with other anthropometric indices (*P* > 0.05).

**Conclusion:**

The quality of dietary fats was associated with some anthropometric indices. Further large-scale studies are required to highlight the importance of dietary fat quality indices in relation to cardio-metabolic risk factors in pediatric age groups. Reducing intake of saturated fatty acids, increasing consumption of monounsaturated fatty acids and a balanced intake of omega-3 and omega-6 to reduce the risk of cardiovascular diseases risk factors are recommended.

## Introduction

Nowadays, weight disorders are one of the worldwide public health priorities in children and adolescents that they have adverse health consequences. According to world health organization (WHO) report, over 340 million children and adolescents (5–19 years) were overweight or obese in 2016 [1]. Evidence has shown a rapid growing prevalence of weight disorders especially overweight and obesity in childhood and adolescent in developing countries as well as in Iran [2]. A recent meta-analysis study estimated the overall prevalence of overweight and obesity about 12 and 11%, respectively in Iran [3]. Excess weight in childhood is associated with increased risk for developing Non-communicable diseases (NCDs) such as cancer, diabetes and cardiovascular and metabolic disorders in adulthood [4]. Various factors including genetic, environmental factors and lifestyle such as diet, have been linked to overweight and obesity [5, 6]. Dietary fat intake is the most energy-dense macronutrient in comparison with protein or carbohydrate and it seems that it is an important predictor of obesity [7]. It has been shown that anthropometric indices are closely related to fat intake in adults, children and adolescents [8–11].

Different anthropometric indicators are used to determine general obesity and abdominal obesity. Body mass index (BMI) is a common anthropometric indicator for assessment of weight status in adults and children. However, BMI would not adequately estimate body composition especially in pediatric age groups [12]. Tri-ponderal mass index (TMI) is calculated as the ratio of body weight and height (kg/m3), indicates excess body fat more accurately than BMI [13]. The neck circumference (NC) is considered as a measure of subcutaneous fat distribution [14]. Study on pediatric age groups showed that NC was associated with high blood pressure, low HDL-C and increased BMI [15] ABSI (A Body Shape index) is an independent anthropometric index that has significant associations with cardiometabolic risk markers in overweight and obese children [16]. Some anthropometric indices including waist circumference (WC), waist-to-hip ratio (WHR), and waist-to-height ratio (WHtR) are useful for determining abdominal obesity .The wrist circumference (WrC) is another simple anthropometric indicator that is easily measured and is associated with metabolic disorders [17].

There are inconsistence findings related to the association between fat intake and obesity in children and adolescents [18, 19]. Studies showed that the relative proportions of fatty acids were more associated with excess weight and NCDs in comparison with their absolute amounts in the diet.

Previous studies suggested some indices of dietary fat quality including the Lipophilic index (LI) and thrombogenic index (TI) that were associated with NCDs [20, 21]. TI considers the ratio of subclasses of SFAs (Saturated fatty acids), MUFAs (Monounsaturated fatty acids) and PUFAs (Polyunsaturated fatty acids). LI considers individual fatty acid levels and their melting points. As a simple method for determining membrane fluidity, it has been demonstrated that it mainly depends on the length of hydrocarbon chains and the number of double bonds found in fatty acids [22].

Studies showed that LI and TI as dietary fat quality indices were associated with NCDs in adults [23, 24]. However, there are few studies about dietary fat quality indices in children and adolescents and it is not yet clear whether these indices are associated with weight disorders in pediatric age groups.

To the best of our knowledge, there is not any study that assess the relationship between dietary LI and TI as dietary fat quality indices with anthropometric measurements in children and adolescents. The present study was conducted to evaluate the quality of the dietary fatty acids intake and its association with different anthropometric measures in Iranian children and adolescents.

## Methods

### Study design

**Fig. 1:**
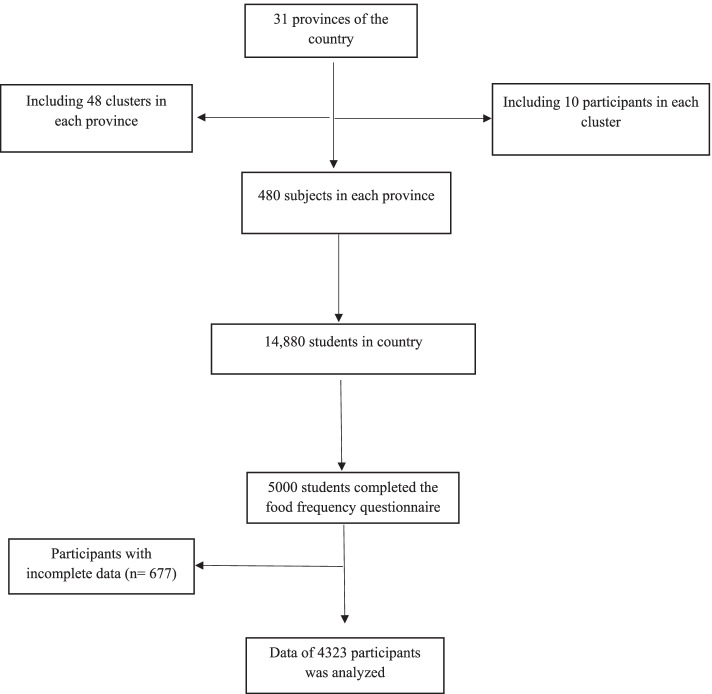
The flowchart of participants’ recruitment

The present study was conducted on 4323 students aged 6-18 years that were selected by multistage cluster sampling from 31 provinces of Iran This nationwide cross-sectional study was performed within the framework of the weight disorders survey of the national survey of school students’ high-risk behaviours” of the fourth phase of school-based surveillance system entitled Childhood and Adolescence Surveillance and Prevention of Adult Non-communicable disease (CASPIAN-IV) [25]. The study protocol was approved by the Ethics Committee of the Isfahan University of Medical Sciences, Isfahan, Iran and Islamic Azad University, Science and Research Branch, Tehran, Iran. The informed oral assent and written consent form were collected by students and parents respectively after description of the aims and protocol of the study. The flowchart of participants’ recruitment is shown in figure 1.

### Assessment of anthropometric measures

Trained health care professionals collected all anthropometric measurements in the selected schools. All schools have a health care provider who monitors students' health throughout the year. The same training courses were held for health care providers in all provinces by the Ministry of Health and Medical Education. Nutritionists were responsible for teaching health care providers. They taught all health care providers how to measure anthropometric measurements accurately and uniformly. A team of trained health care experts performed measurements under standard protocols by calibrated instruments.

Body weight was measured using a Seca scale (SECA, Ham-burg, Germany) to the nearest 0.1 kg with light clothing and without shoes. Height was measured using a Seca stadiometer (SECA, Ham-burg, Germany) to the nearest 0.1 centimeter without shoes. Waist circumference (WC) was measured using a non-elastic tape (Seca, non elastic tape, Germany ) at a point midway between the lower border of the rib cage and the iliac crest at the end of normal expiration to the nearest 0.1 cm. Hip circumference (HC) was measured at the widest part of the hip at the level of the greater trochanter. Wrist circumference was measured to the nearest 0.1 cm on the dominant arm using a tape meter. Participants were asked to hold their arm on a flat surface such as a table. The superior border of the tape measure was placed just distal to the prominences of radial and ulnar bones. The neck circumference (NC) was measured under the Adam's apple in a comfortable position.

Body mass index (BMI) was calculated by dividing body weight (kg) to the height squared (m^2^). The following cut-off points for age and gender were used to classification BMI as underweight (<5th percentile), normal-weight (5-84^th^ percentile), over-weight (85-94th percentile) and obese (>95th percentile) [26]. The tri-ponderal mass index (TMI) was calculated by the ratio of weight in kilograms to height in meters at the third power (weight (kg) / height (m^3^)) [27]. A body shape index (ABSI) was based on waist circumference (WC) adjusted for height and weight using the following formula [28]:

$$\mathrm{ABSI}=\mathrm{WC}\left(\mathrm m\right)/\left[\mathrm{BMI}^{2/3}\times\mathrm{height}\left(\mathrm m\right)^{1/2}\right]$$

Waist to hip ratio (WHR) and waist to height ration (WHtR) were calculated by dividing WC to HC and WC to height, respectively.

### Physical activity (PA) assessment

To measure the level of PA in adolescents and children, the Physical Activity Questionnaire for Adolescents (PAQ-A) was used [29]. Sufficient PA was defined as doing exercises at least 30 minutes per day that led to sweating and increasing in respiratory or heart rate (response options ranged from 0 to 7 days); PA less than 30min per day was defined as low.

### Socio-economic status

Socioeconomic status (SES) of students was determined by performing a principal components analysis. Relevant questions that were entered in analysis were type of home, parental education, parent’s job, having private car and computer, and type of student’s school (private, public). SES was divided into tertiles including low, moderate and high [30].

### Dietary assessment

Food frequency questionnaire (FFQ) of 168 items was used to collect information regarding usual dietary intake in 4323 samples. This questionnaire was designed and validated previously [31]. Amount of each food item was converted into gram using household scale guide and absolute quantities of dietary fatty acids were determined using standard portion sizes of food items from the United States Department of Agriculture Food Composition Databases [32]. The Japanese Lipid Bank database [33] was used for determination of melting points of dietary fatty acids.

### Thrombogenic index (TI) formula

Thrombogenic index (TI) = [(14:0+ 16:0 + 18:0) / [(0.5ΣMUFA)] + (0.5Σω-6 PUFA) + (3 Σω-3 PUFA) + (Σ ω-3 PUFA / Σω-6 PUFA)] [30] ; where 14:0 = myristic acid, 16:0 = palmitic acid, 18:0 = stearic acid, MUFA = monounsaturated fatty acids, Σω-6 PUFA = sum of omega-6 polyunsaturated fatty acids, Σω-3 PUFA = sum of omega-3 polyunsaturated fatty acids.

### Lipophilic index (LI) formula

The lipophilic index of dietary intake was computed by multiplying the intake of each fatty acid (in grams) by its specific melting point (°C), the sum of these two items, and then dividing by the total of fatty acid intake (in grams). Dietary lipophilic index (LI) = Σ [Fatty acid (g) i × Melting point (°C)i] / ΣFatty acid (g) i [22].

High dietary TI and LI are correlated with higher intakes of saturated and trans fatty acids and lower intakes of PUFAs and MUFAs. So, lower indices are considered protective. Higher TI and LI as the quality of dietary fats indicators could increase the risk of obesity-related cardiovascular diseases.

### Covariate for adjustment

Age, sex, place of residence, SES and PA were considered as covariate. Different age rage (6-18 years), two genders and 31 different provinces can affect the results. Participants lived in urban and rural areas and had various SES and PA. These factors can affect the main relationship and should be adjusted.

### Statistical analysis

Mean±standard deviation (SD) and frequency (percentage) were used to illustrate quantitative and qualitative variables, respectively. Normality of data was assessed graphically. To compare mean values of the anthropometric indices across the TI and LI quartiles, one-way analysis of variance was conducted. The percentages of categorical variables were compared using Pearson’s χ^2^ test. For evaluating the association between anthropometric indices (dependent variables) and TI and LI score (categorized independent variables), linear regression models were used. There was no adjustment for covariates in one of the models (model I), while age, sex, place of residence, SES and PA were adjusted in the other model (model II). The first quartile of indices was considered as the reference. Multicollinearity between covariates was assessed by variance inflation factor (VIF), as a rule of thumb, a variable whose VIF values are greater than 10 could be considered as a linear combination of other independent variables. Observed power to detect significant association of anthropometrics measures with TI and LI indices was also calculated. All statistical analysis was performed using SPSS 23 (SPSS, IBM, USA). *P*-value less than 0.05 was considered as statistically significant.

## Results

In this national survey, 4323 subjects (52.51% boys) with mean (SD) age of 11.39 (3.19) years were included. Most participants lived in urban areas (71.3%). Urban residence significantly had a higher level of IT and LI compared with rural residence (*P* < 0.05). Older children had higher levels of TI and LI (*P* < 0.05). There was no significant association between sex and TI (*P* = 0.72) or LI levels (*P* = 0.81).

Table 1 shows the participants’ characteristics according to the TI quartiles. The anthropometric measures including height, wrist, HC, and NC were significantly lower among participants in the upper TI quartile compared with lower quartile (*p* < 0.05). Inversely the participants in the upper TI quartiles had significantly higher height z-scores in comparison to those in the lower quartiles. Individuals in the fourth quartile of TI index had higher PA levels compared with those in the first quartile (44.89 ± 11.72 *vs*. 42.60 ± 13.54 Met-h/w, *P* < 0.001). Mean TI value for the total population was 1.06 ± 0.31 mEq/day.

**Table 1:** Characteristics of the study population according to quartiles of TI

		TI Quartiles					
		Q1	Q2	Q3	Q4	Total	*p*-value
Number		1080	1081	1081	1081	4323	
Gender, Number (%)	boy	576(25.4)^a^	569(25.1)	552(24.3)	573(25.2)	2270	0.726
	girl	504(24.5)	512(24.9)	529(25.8)	508(24.7)	2053	
Region, Number (%)	rural	362(29.2)	327(26.3)	285(23.0)	267(21.5)	1241	**< 0.001**
	urban	718(23.3)	754(24.5)	796(25.8)	814(26.4)	3082	
Age (years)		11.70 ± 3.21^b^	11.44 ± 3.11	11.38 ± 3.22	11.06 ± 3.22	11.39 ± 3.19803	**< 0.001**
PA		42.60 ± 13.54	44.25 ± 12.91	43.79 ± 11.80	44.89 ± 11.72	43.88 ± 12.53977	**< 0.001**
LI (mEq/day)		22.19 ± 2.11	25.22 ± 1.33	27.27 ± 1.11	30.41 ± 1.84	26.27 ± 3.41752	**< 0.001**
TI (mEq/day)		0.70 ± 0.10	0.93 ± 0.05	1.12 ± 0.06	1.48 ± 0.22	1.06 ± .31282	**< 0.001**
BMI Z-score		-0.04 ± 0.95	-0.04 ± 0.92	0.05 ± 1.06	0.02 ± 1.04	0.00 ± .99708	0.076
Weight Z-score		-0.02 ± 1.02	-0.04 ± 0.94	0.03 ± 1.03	0.03 ± 1.01	0.00 ± .99710	0.283
Height Z-score		-0.15 ± 0.89	-0.09 ± 1.04	-0.12 ± 0.97	-0.03 ± 1.18	-0.09 ± 1.02442	**0.038**
Wrist Z-score		0.04 ± 1.08	-0.04 ± 0.94	-0.01 ± 1.00	0.00 ± 0.97	0.00 ± 1.00	0.306
NC Z-score		0.06 ± 1.03	-0.04 ± 0.96	0.01 ± 1.01	-0.03 ± 0.98	0.00 ± 1.00	0.098
WC Z-score		0.00 ± 1.07	-0.03 ± 0.94	0.01 ± 1.01	0.03 ± 0.97	0.00 ± 1.00	0.594
HC Z-score		0.01 ± 1.03	-0.04 ± 0.97	0.02 ± 0.98	0.01 ± 1.01	0.00 ± 1.00	0.47
WHR (cm)		0.83 ± 0.11	0.83 ± 0.13	0.82 ± 0.08	0.83 ± 0.09	0.83 ± .10668	0.27
WHtR (cm)		0.45 ± 0.07	0.45 ± 0.06	0.46 ± 0.07	0.46 ± 0.07	0.45 ± .06468	0.372
TMI (kg/m3)		12.87 ± 3.13	12.87 ± 4.17	13.28 ± 4.96	13.20 ± 5.70	13.06 ± 4.59	0.069
TMI Z-score		-0.03 ± 0.89	-0.03 ± 0.98	0.04 ± 1.05	0.02 ± 1.06	0.00 ± 1.00	0.29
ABSI		0.79 ± 0.09	0.78 ± 0.08	0.78 ± 0.09	0.79 ± 0.08	0.78 ± 0.08	0.285

*TI* thrombogenic index, *BMI* body mass index, *WC* waist circumference, *HC* hip circumference, *NC* neck circumference, *WHR* waist to hip ratio, *WHtR* waist to height ratio, *PA* physical activity, *TMI*, tri-ponderal mass index, *ABSI* a body shape index

^a^ Number (%), ^b^ mean±SD. *P* values for the comparisons between quantitative variables were derived using the ANOVA, while for the comparisons of categorical variables the χ 2 test was used

Table 2 presents the comparison of participants’ characteristics across the quartiles of dietary LI. In the fourth quartile of LI, weight, height, wrist circumference, HC, and NC were lower and height z-score was higher than those in the first quartile. However, individuals in the fourth quartile of LI had higher PA levels compared with those in the first quartiles (44.72 ± 12.03 *vs*. 42.28 ± 13.93 Met-h/w, *P* < 0.001). Mean LI value for the total population was 26.27 ± 3.42 mEq/day.

**Table 2:** Characteristics of the study population according to quartiles of LI

		LI Quartiles					
		Q1	Q2	Q3	Q4	Total	*p*-value
Number		1080	1081	1081	1081	4323	
Gender, Number (%)	boy	559(24.6) a	575(25.3)	561(24.7)	575(25.3)	2270	0.81
	girl	521(25.4)	506(24.6)	520(25.3)	506(24.6)	2053	
Region, Number (%)	rural	395(31.8)	324(26.1)	281(22.6)	241(19.4)	1241	**< 0.001**
	urban	685(22.2)	757(24.6)	800(26.0)	840(27.3)	3082	
Age (years)		11.85 ± 3.19b	11.42 ± 3.11	11.24 ± 3.19	11.05 ± 3.24	11.39 ± 3.20	**< 0.001**
PA		42.28 ± 13.93	44.10 ± 12.43	44.43 ± 11.52	44.72 ± 12.03	43.88 ± 12.54	**< 0.001**
LI (mEq/day)		21.92 ± 1.74	25.20 ± 0.68	27.36 ± 0.62	30.61 ± 1.66	26.27 ± 3.42	**< 0.001**
TI (mEq/day)		0.72 ± 0.12	0.94 ± 0.10	1.12 ± 0.11	1.46 ± 0.25	1.06 ± 0.31	**< 0.001**
BMI Z-score		-0.05 ± 0.95	-0.02 ± 0.96	0.02 ± 1.01	0.05 ± 1.06	0.00 ± 1.00	0.102
Weight Z-score		-0.05 ± 1.02	0.00 ± 0.95	0.03 ± 1.02	0.03 ± 1.00	0.00 ± 1.00	0.211
Height Z-score		-0.18 ± 0.88	-0.05 ± 1.06	-0.08 ± 0.94	-0.07 ± 1.19	-0.09 ± 1.02	**0.009**
Wrist Z-score		0.02 ± 1.09	-0.01 ± 0.96	-0.02 ± 0.98	0.01 ± 0.95	0.00 ± 1.00	0.862
NC Z-score		0.06 ± 1.03	-0.03 ± 0.98	-0.01 ± 0.96	-0.03 ± 1.01	0.00 ± 1.00	0.094
WC Z-score		-0.03 ± 1.05	-0.01 ± 0.98	0.01 ± 0.99	0.03 ± 0.97	0.00 ± 1.00	0.456
HC Z-score		-0.03 ± 1.04	-0.01 ± 0.97	0.02 ± 0.97	0.02 ± 1.01	0.00 ± 1.00	0.585
WHR (cm)		0.83 ± 0.12	0.83 ± 0.13	0.83 ± 0.08	0.83 ± 0.08	0.83 ± 0.11	0.53
WHtR (cm)		0.45 ± 0.06	0.45 ± 0.06	0.45 ± 0.07	0.46 ± 0.07	0.45 ± 0.06	0.342
TMI Z-score		-0.03 ± 0.89	-0.02 ± 1.04	0.00 ± 0.96	0.05 ± 1.09	0.00 ± 1.00	0.296
ABSI		0.78 ± 0.09	0.79 ± 0.08	0.78 ± 0.08	0.79 ± 0.08	0.78 ± 0.08	0.603

*LI* lipophilic index, *BMI* body mass index, *WC* waist circumference, *HC* hip circumference, *NC* neck circumference, *WHR* waist to hip ratio, *WHtR* waist to height ratio, *PA* physical activity, *TMI* tri-ponderal mass index, *ABSI* a body shape index

^a^ Number (%), ^b^ mean ± SD. *P* values for the comparisons between quantitative variables were derived using the ANOVA, while for the comparisons of categorical variables the χ 2 test was used

Table 3 shows mean of fatty acids intake among total population and genders. In addition, it shows the association of different fatty acids intake with TI and LI scores.

**Table 3:** Fatty acids intakes

		Fatty acids intake (mean ± SD)			Correlation (r) ^a^ with TI	Correlation (r) ^a^ with LI
	melting point	girl	boy	total		
Animal _Fat		44.36 ± 24.14	42.83 ± 23.57	43.56 ± 23.85	.169^b^	.131^b^
Vegetable _Fat		34.88 ± 19.12	33.67 ± 18.36	34.25 ± 18.73	-.394^b^	-.392^b^
Total _Fat		85.38 ± 35.65	82.62 ± 34.23	83.93 ± 34.94	-.092^b^	-.111^b^
**SFAs**		29.60 ± 13.03	28.91 ± 13.05	29.24 ± 13.04	.339^b^	.318^b^
c4_00	-7.9	0.95 ± 0.57	0.91 ± 0.56	0.93 ± 0.56	.559^b^	.490^b^
c6_00	-3.4	0.63 ± 0.37	0.61 ± 0.36	0.62 ± 0.37	.569^b^	.502^b^
c8_00	16.7	0.50 ± 0.27	0.49 ± 0.27	0.49 ± 0.27	.562^b^	.508^b^
c10_00	31.6	1.11 ± 0.61	1.08 ± 0.60	1.09 ± 0.60	.522^b^	.473^b^
c12_00	44.2	1.31 ± 0.76	1.27 ± 0.78	1.29 ± 0.77	.403^b^	.425^b^
c14_00	53.9	3.41 ± 1.78	3.29 ± 1.77	3.35 ± 1.78	.563^b^	.515^b^
c16_00	63.1	15.26 ± 6.40	14.76 ± 6.34	15.00 ± 6.37	.317^b^	.277^b^
c18_00	69.6	6.71 ± 3.00	6.58 ± 3.03	6.64 ± 3.01	.245^b^	.230^b^
**MUFAs**		29.31 ± 13.36	28.36 ± 12.88	28.81 ± 13.12	-.223^b^	-.190^b^
c16_01	0	0.95 ± 0.47	0.92 ± 0.48	0.94 ± 0.48	.278^b^	.278^b^
c18_01	16	28.99 ± 13.31	27.90 ± 12.74	28.42 ± 13.02	-.213^b^	-.188^b^
c20_01	23.25	0.19 ± 0.13	0.19 ± 0.14	0.19 ± 0.13	-.384^b^	-.343^b^
c22_01	33.35	0.01 ± 0.02	0.01 ± 0.03	0.01 ± 0.02	-.134^b^	-.077^b^
**PUFAs**		19.77 ± 11.02	18.77 ± 9.52	19.24 ± 10.27	-.422^b^	-.507^b^
c18_02	-5	17.96 ± 9.99	17.16 ± 8.66	17.54 ± 9.32	-.432^b^	-.509^b^
c18_3n_3	-11.15	0.32 ± 0.46	0.28 ± 0.25	0.30 ± 0.37	-.238^b^	-.244^b^
c18_3n_6	-11.15	0.01 ± 0.01	0.01 ± 0.01	0.01 ± 0.01	-.231^b^	-.230^b^
c18_03	-11.15	1.58 ± 0.81	1.48 ± 0.68	1.52 ± 0.75	-.057^b^	-.127^b^
c18_04	-57	0.03 ± 0.06	0.03 ± 0.07	0.03 ± 0.06	-.100^b^	-.097^b^
c20_04	-49.5	0.16 ± 0.10	0.15 ± 0.10	0.16 ± 0.10	.017	.037^c^
EPA	-54.1	0.06 ± 0.12	0.06 ± 0.14	0.06 ± 0.13	-.094^b^	-.090^b^
DPA	-78	0.01 ± 0.02	0.01 ± 0.02	0.01 ± 0.02	.031^c^	.055^b^
DHA	-44.15	0.10 ± 0.15	0.10 ± 0.17	0.10 ± 0.16	-.096^b^	-.091^b^
**TFAs**		0.92 ± 0.50	0.88 ± 0.48	0.90 ± 0.49	.147^b^	.087^b^
F_16_1lt	31	0.00 ± 0.00	0.00 ± 0.00	0.00 ± 0.00	-.049^b^	-.010
F_18_1LT	48.7	1.67 ± 2.30	1.77 ± 2.42	1.72 ± 2.36	-.078^b^	.134^b^
F_18_2trans	5.7	0.16 ± 0.14	0.17 ± 0.14	0.17 ± 0.14	.006	.183^b^
Chol		318.72 ± 220.70	328.06 ± 225.45	323.63 ± 223.23	.166^b^	.177^b^

*MUFA* monounsaturated fatty acid, *PUFA* polyunsaturated fatty acid, *SFA* saturated fatty acid, *TFA* Trans fatty acid

^a^ Pearson’s correlation. ^b^ significant in 0.01 level, ^c^ significant in 0.05 level

Dietary LI and TI were positively correlated with SFAs, including 4:0, 6:0, 8:0, 10:0, 12:0, 14:0, 16:0, 18:0 and inversely correlated with PUFAs, including c18_02, c18_3n_3, c18_3n_6, c18_03, c18_04, eicosapentaenoic acid (EPA), docosahexaenoic acid (DHA) and directly correlated with c20_04, docosapentaenoic acid (DPA). There was inverse association between dietary LI, TI and MUFA including c18_01, c20_01, c22_01 and direct correlation between dietary LI, TI and c16_01. Also, LI was positively correlated with total TFA including18:1, 16:1 and 18:2.

Results of linear regression for the associations between TI and anthropometric measures are shown in Table 4. There was a positive association between BMI z-score and TI. In the crude model, individuals in the third quartile of TI had higher BMI z-score compared with those in the first quartile (β = 0.09, 95% CI: 0.01, 0.18). However, the association remained marginally significant in the adjusted model (*P* = 0.062). Also, individuals in the fourth quartile of TI had higher height z-score compared with those in first quartile (0.121; 95% CI: - 0.03, 0.21) in the crude model. But, when the potential confounders were considered, these relationships disappeared. There was an inverse association between NC z-score and quartiles of TI (β = 0.11, 95% CI: 0.01, *p* = 0.013). There was no association between TI and other anthropometric indices. VIF as a measure representing degree of multicollinearity was less than 5 for all multivariate models. Observed powers to detect significant association between TI and different anthropometrics measures in type one error of 0.05 were ranged 24.0 to 68.6%.

**Table 4:** Associations between the TI and anthropometric measures

	Model I					Model II				
	TI quartiles					TI quartiles				
	Q1	Q2	Q3	Q4	R^2^, BIC	Q1	Q2	Q3	Q4	R^2^, BIC
BMI Z-score	Ref.	-0.003(0.09,0.08)^a^	0.09(0.01,0.18)	0.06(-0.03,0.14)	0.001	Ref.	-0.01(-0.09,0.08)	0.08(-0.004,0.16)	0.05(-0.04,0.13)	0.0007
		0.953^b^	**0.034**	0.17	12169.2		0.874	0.062	0.294	12183.6
Weight Z-score	Ref.	-.013(-0.10,0.07)	.053(-0.03,0.14)	.051(-0.03,0.14)	0.0002	Ref.	-0.02(-0.11,0.06)	0.03(-0.05,0.11)	0.02(-0.06,0.10)	-0.0004
		0.768	0.213	0.235	12257.5		0.57	0.484	0.638	12282.3
Height Z-score	Ref.	.061(-0.02,0.15)	.026(-0.06,0.11)	.121(0.03,0.21)	0.0013	Ref.	0.04(-0.04,0.13)	-0.002(0.09,0.08)	0.07(-0.01,0.16)	0.0195
		0.163	0.553	**0.006**	12483.5		0.342	0.971	0.1	12426.2
WC Z-score	Ref.	-0.03(-0.11,0.06)	0.01(-0.07,0.10)	0.03(-0.05,0.12)	0.0004	Ref.	-0.04(-0.12,0.05)	-0.01(-0.10,0.07)	0.002(-0.08,0.09)	0.0003
		0.538	0.801	0.462	12233.9		0.402	0.775	0.957	12253.5
Wrist Z-score	Ref.	-0.08(-0.16,0.00)	-0.05(-0.14,0.03)	-0.04(-0.12,0.04)	0.0001	Ref.	-0.09(-0.17,0.003)	-0.06(-0.15,0.02)	-0.06(-0.14,0.03)	-0.0005
		0.06	0.236	0.346	12209.6		**0.042**	0.145	0.191	12234.7
NC Z-score	Ref.	-0.10(-0.18,-0.01)	-0.05(-0.14,0.03)	-0.09(-0.17,-0.01)	0.0008	Ref.	-0.10(-0.19,-0.02)	-0.07(-0.15,0.02)	-0.11(-0.19,-0.02)	0.0002
		**0.026**	0.234	**0.038**	12132.9		**0.017**	0.132	**0.013**	12157.4
HC Z-score	Ref.	-0.05(-0.14,0.03)	0.01(-0.07,0.10)	-0.002(0.09,0.08)	0.0006	Ref.	-0.06(-0.15,0.02)	-0.02(-0.10,0.07)	-0.04(-0.12,0.05)	-0.0004
		0.229	0.797	0.962	12241.7		0.134	0.719	0.397	12265.36
WHR	Ref.	0.005(-0.004,0.01)	-0.001(-0.01,0.01)	0.01(-0.003,0.02)	0.0002	Ref.	0.004(0.00,0.01)	-0.002(0.01,0.01)	0.004(-0.01,0.01)	0.06
		0.298	0.783	0.158	-7021.6		0.36	0.736	0.371	-7255
WHtR	Ref.	-0.003(0.01,0.002)	0.001(-0.004,0.01)	0.001(0.004,0.01)	0.0007	Ref.	-.003(-0.01,0.002)	.001(-0.01,0.01)	-0.0001(0.01,0.01)	0.004
		0.275	0.64	0.681	-11322.8		0.236	0.843	0.987	-11319.2
TMI Z-score	Ref.	0.0004(-0.08,0.08)	0.07(-0.02,0.15)	0.05(-0.04,0.13)	0.0002	Ref.	-0.0004(0.08,0.08)	0.06(-0.02,0.15)	0.04(-0.04,0.13)	-0.0001
		0.993	0.119	0.277	12197.9		0.992	0.138	0.315	12221.2
ABSI	Ref.	0.0002(-0.01,0.01)	-0.004(0.01,0.004)	0.004(0.004,0.01)	0.0002	Ref.	-0.002(-0.01,0.01)	-0.01(0.01,0.002)	-0.001(-0.01,0.01)	0.07
		0.952	0.334	0.327	-9014.4		0.62	0.132	0.861	-9298.9

*TI* thrombogenic index, *BMI* body mass index, *WC* waist circumference, *HC* hip circumference, *NC* neck circumference, *WHR* waist to hip ratio, *WHtR* waist to height ratio, *TMI* tri-ponderal mass index, *ABSI* a body shape index, *R*^2^ adjusted R-square, *BIC* Bayesian information criteria

^a^ Coefficient regression (95% CI) in a linear model regression, ^b^ *p*-value. Model I: crude model, Model II: Adjusted for age, gender, physical activity, residence area

Table 5 shows the association between LI and anthropometric measures. There was a direct association between BMI z-score and LI. In the crude model, individuals in the fourth quartile of LI had higher BMI z-score compared with those in the first quartile (*β *= 0.10, 95% CI: 0.01, 0.18). However, the association disappeared in the adjusted model (*P* = 0.087). Significant associations were found in both crude (β = 0.10, 95% CI: 0.01, *p* = 0.025) and adjusted models (β = 0.12, 95% CI: 0.01, *p* = 0.009) between height z-score and quartiles of LI (*p* < 0.05). However, there was an inverse association between NC z-score and quartiles of LI (β = 0.12, 95% CI: 0.01, *p* = 0.006). There was no association between LI and other anthropometric indices. VIF as a measure representing degree of multicollinearity was less than 5 for all multivariate models. Observed powers to detect significant association between LI and different anthropometrics measures in type one error of 0.05 were ranged 9.8 to 82.3%.

**Table 5:** Associations between the LI index and anthropometric measures

	Model I					Model II				
	LI quartiles					LI quartiles				
	Q1	Q2	Q3	Q4	R^2^, BIC	Q1	Q2	Q3	Q4	R^2^, BIC
BMI Z-score	Ref	0.03(-0.05,0.11)	0.08(-0.01,0.16)	0.10(0.01,0.18)	0.001	Ref	0.02(-0.06,0.10)	0.06(-0.03,0.14)	0.07(-0.01,0.16)	0.001
		0.481	0.078	**0.024**	12161.5		0.638	0.17	0.087	12184.6
Weight Z-score	Ref	0.05(-0.04,0.13)	0.08(-0.01,0.16)	0.08(0.00,0.16)	0.0004	Ref	0.03(-0.06,0.11)	0.04(-0.04,0.13)	0.03(-0.05,0.12)	-0.0003
		0.258	0.071	0.061	12256.8		0.546	0.331	0.461	12281.5
Height Z-score	Ref	0.14(0.05,0.22)	0.11(0.02,0.19)	0.12(0.03,0.20)	0.002	Ref	0.10(0.01,0.18)	0.05(-0.03,0.14)	0.04(-0.04,0.13)	0.02
		**0.002**	**0.015**	**0.009**	12480.4		**0.025**	0.211	0.314	12424.2
WC Z-score	Ref	0.03(-0.06,0.11)	0.04(-0.04,0.12)	0.07(-0.02,0.15)	0.0006	Ref	0.01(-0.08,0.09)	0.005(-0.08,0.09)	0.02(-0.06,0.10)	0.001
		0.526	0.351	0.112	12233.2		0.895	0.908	0.65	12252.5
Wrist Z-score	Ref	-0.02(-0.11,0.06)	-0.04(-0.12,0.05)	-0.01(-0.10,0.07)	0.0002	Ref	-0.04(-0.12,0.05)	-0.05(-0.14,0.03)	-0.04(-0.12,0.05)	-0.001
		0.577	0.409	0.761	12212.5		0.404	0.213	0.386	12237.6
NC Z-score	Ref	-0.10(-0.18,-0.01)	-0.07(-0.16,0.01)	-0.09(-0.18,-0.01)	0.001	Ref	-0.11(-0.20,-0.03)	-0.09(-0.18,-0.01)	-0.12(-0.21,-0.04)	0.0003
		**0.024**	0.098	**0.036**	12132.9		**0.01**	**0.031**	**0.006**	12157.3
HC Z-score	Ref	0.02(-0.06,0.10)	0.05(-0.04,0.13)	0.05(-0.03,0.14)	0.0004	Ref	-0.004(-0.09,0.08)	0.01(-0.07,0.09)	-0.003(-0.09,0.08)	-0.001
		0.629	0.255	0.234	12242.4		0.92	0.833	0.938	12265.7
WHR	Ref	0.004(-0.01,0.01)	0.0003(0.01,0.01)	0.01(0.00,0.01)	0.0005	Ref	0.001(-0.01,0.01)	-0.002(0.01,0.01)	0.002(-0.01,0.01)	0.01
		0.422	0.946	0.206	-7019.9		0.759	0.624	0.669	-7253.6
WHtR	Ref	-0.001(0.01,0.005)	0.001(0.004,0.01)	0.004(0.001,0.01)	0.0001	Ref	-0.002(0.01,0.004)	0.0001(0.01,0.01)	0.002(0.003,0.01)	0.004
		0.814	0.601	0.147	-11323.1		0.787	0.416	0.739	-11319.3
TMI Z-score	Ref	0.01(-0.07,0.10)	0.02(-0.06,0.11)	0.08(-0.01,0.16)	0.0002	Ref	0.01(-0.08,0.09)	0.02(-0.06,0.11)	0.07(-0.02,0.16)	-0.0001
		0.787	0.562	0.075	12198.04		0.828	0.633	0.107	12221.2
ABSI	Ref	0.002(0.005,0.01)	0.001(-0.01,0.01)	0.005(0.002,0.01)	0.0004	Ref	-0.001(-0.01,0.01)	-0.003(0.01,0.00)	-0.001(0.01,0.01)	0.07
		0.528	0.69	0.184	-9012.5		0.578	0.976	0.471	-9296.9

*LI* lipophilic index, *BMI* body mass index, *WC* waist circumference, *HC* hip circumference, *NC* neck circumference, *WHR* waist to hip ratio, *WHtR* waist to height ratio, *TMI* tri-ponderal mass index, *ABSI* a body shape index, *R*^2^ adjusted R-square, *BIC* Bayesian information criteria

^a^ Coefficient regression (95% CI) in a linear model regression, ^b^*p*-value. Model I: crude model, Model II: Adjusted for age, gender, physical activity, residence area

## Discussion

To the best of our knowledge, this is the first study that evaluated the relationship between LI and TI with anthropometric measures in a large nationwide sample among Iranian children and adolescents. We found an association between higher LI and TI and lower NC Z-score. There was a positive association between LI and height Z-score. We also observed that LI and TI were directly associated with BMI Z-score in the crude model. However, there was no significant association between LI and TI with other anthropometric indices including weight Z-score, WC Z-score, wrist circumference Z-score, HC Z-score, WHR, WHtR, TMI Z-score and ABSI.

High dietary LI and TI were correlated with higher intake of SFAs, cholesterol and trans fatty acids and lower intake of PUFAs and MUFAs. LI and TI as dietary fats quality indicators could increase the risk factors of NCDs [34].

The results of the present study indicated that the LI and TI were positively associated with BMI z-score. The results of our study were similar to previous studies. A cross-sectional study among 295 women aged 18–59 years in Ghana, reported a direct association between LI and TI with BMI [23]. The Women’s Health Initiative (WHI) observational cohort study, demonstrated an association between higher dietary LI (median (IQR) 27.6 (3.5)) and higher BMI among postmenopausal women with coronary heart disease (CHD) [35]. Study on 504 participants with age range of 18–64 years in Iran, demonstrated a positive association between LI (mean (SD) 34.99(6.91)) and BMI (β = 0.17; *P *= 0.04) [36]. A prospective nested case-control study among US men with CHD, reported that both plasma and erythrocyte LI were consistently correlated with higher BMI [37]. Fatty acid contents may change with different diet, so measurement of fatty acid composition in plasma or erythrocyte may not represent long-term average values [38]. One study investigated the association between the adipose tissue LI and the occurrence of total ischemic stroke among 57,053 subjects aged 50–64 years in Denmark. Results demonstrated that who had the lowest LI were more likely to have a higher BMI than the subjects with higher LI [39]. Interpreting LI depends upon the type of biological origin. LI of adipose tissue reflects dietary fatty acid composition rather than fluidity of cell membranes.

According to our findings, we found an inverse association between LI and TI with NC Z-score. Studies showed that NC was correlated with cardio-metabolic risk factors [15]. A cross-sectional survey among Chinese postmenopausal women, revealed that NC was significantly correlated with HOMA-IR and triglycerides, and negatively correlated with HDL-C [40]. Another study evaluated the association between NC and metabolic risk factors in obese children between 5 and 18 years of age. They showed NC was strongly correlated with increased insulin levels and lipid metabolism disorders (elevated triglyceride and decreased HDL) [41]. It showed that subcutaneous fat in the upper body released more systemic free fatty acids and is more lipolytically active than lower body adipose tissue [42]. Large NC means excessive accumulation of subcutaneous fat in the neck, which contributes to a greater flux of the free fatty acid release into the circulation. Subsequently, elevated free fatty acid contributes to increase synthesis and ectopic deposition of triglycerides, insulin resistance, and inflammation [43]. Thus, NC as a proxy of neck subcutaneous fat has diagnostic value for evaluating metabolic disturbances.

In the present study, a direct association was found between LI and height Z-score. There is no study that has reported the association of dietary fat quality with height Z-score in children and adolescents. A cross-sectional study was conducted among 307 children aged 2-6 years in Ghana. The findings indicated that n-6 fatty acids were significantly positively associated with height Z-score and these fatty acids were critical in childhood linear growth [44]. Another study in China between 196 children aged 1-5 years reported that consumption of fatty acids may be associated with growth and height in children aged 1-5 years. Arachidonic acid (AA) and total n-6 fatty acids were the only fatty acids to show significant difference between malnourished and nourished groups in this population. Children with height z-scores less than −2SD consumed more AA than children with height z-scores above −2SD. This implies that more poorly nourished children may have consumed more AA at the time of assessment than better-nourished children. However, essential fatty acids intakes were low in both groups [45].

Results of our study were different with previous studies and found that arachidonic acid had a crucial role in correlation with LI. Thus, high arachidonic acid proportion could be specifically related to childhood and adolescent growth and on the other hand, promote inflammation, endothelial activation, thrombosis and decrease fliuidity membrane [46].

In the present study, there was no association between TI or LI and WHtR Z-score and WC Z-score. However, a cross sectional study, revealed a positive association between TI and WC and WHtR among Ghanaian women. In addition, they observed a positive association between LI and WHtR [23]. Another observational study that was carried out on 504 Iranian population with metabolic phenotypes, indicated positive associations between LI and WC (β = 0.18; *P *= 0.01) [36].

The observed inconsistency between the results of studies can be attributed to differences in study design, population characteristics (age, health status, and geographic differences), and differential effects by type of fatty acids, potential measurement error and bias in study participants.

Several pathways and mechanisms explained the inconsistent results. However, the exact mechanisms of the associations between anthropometric indices and the dietary fat quality indices including TI and LI have not been understood. According to lipophilicity, the quality of fatty acids is determined by their melting points, which correspond to three main characteristics of fatty acids, such as a longer hydrocarbon chain, a higher saturation rating and a presence of a double bond in the trans configuration [47]. Therefore, higher LI is associated with high melting points of fatty acids and may indicate less membrane fluidity [48]. Less membrane fluidity is due to an excessive accumulation of plasma triglycerides [49], and also increase the concentration of circulating cholesterol which can lead to insulin resistance [50].

Higher intake of long-chain SFAs such as myristic, palmitic and stearic acids and high arachidonic acid intake lead to increase values of LI and TI. These fatty acids have proinflammatory and procoagulant effects and can develop atherosclerosis. In addition, arachidonic acid can mediate adipogenesis [51]. SFAs reduce expression of genes involve in fatty acid β-oxidation and triglyceride synthesis. It can decrease lipid handling capacity of the adipocytes and increase inflammation [52]. Higher intake of PUFA-3 and MUFA suppress the expression of lipogenesis genes in various organs and increase fatty acid β-oxidation [53]. Therefore, unsaturated fatty acids can play an important role in the weight loss and body composition [54]. In general, omega-3 fatty acids and MUFA have the anti-inflammatory effect, anti-arrhythmic effect, and anti-thrombotic effect, while omega-6 fatty acids tend to cause inflammation and thrombus formation [55].

The current study has several limitations. First, the amount of some dietary trans fatty acids were negligible and therefore they had little effect on the dietary LI and TI. Because of the important role of trans fatty acids, this might affect the results. Second, LI and TI were derived from dietary fatty acids that were computed from food frequency questionnaires. So, it was prone to measurement error. Finally, because of the cross-sectional design of our study, the causal relationship remained unclear. Despite these limitations, the study has some strengths including large sample size of children and adolescents, the nationwide design of the study, adjusted data by important confounding variables and using new anthropometric indices including TMI and ABSI.

## Conclusion

The findings of the present study showed an inverse association between LI and TI with NC z-score. Also, there was a positive association between LI and height z-score and inverse association between TI and wrist circumference in children and adolescents. Reducing intake of foods rich in arachidonic acid and SFA, a balanced intake of omega-3 and omega-6 PUFA and MUFA are recommended to reduce the risk of developing NCDs. Future studies are needed to determine the specific relationship between dietary fat quality and cardio-metabolic risk factors in pediatric age groups.

## Data Availability

The data that support the findings of this study are available from the corresponding author, upon reasonable request..

## Abbreviations

ABSI: A body shape index
BMI: Body Mass Index
DHA: Docosahexaenoic acid
EPA: Eicosapentaenoic acid
FFQ: food frequency questionnaire
HC: Hip circumference
LI: Lipophilic index
MET-minutes/weeks: Metabolic equivalent minutes per week
MUFA: Monounsaturated fatty acids
NC: Neck circumference
NCDs: Non-communicable diseases
PA: Physical Activity
PAQ-A: Physical Activity Questionnaire for Adolescents
PUFA: Polyunsaturated fatty acids
SDs: standard deviations
SFAs: Saturated fatty acids
SES: Socio-economic status
TI: Thrombogenic index
TMI: Triponderal mass index
WC: Waist circumference
WHR: waist to hip ratio
WHtR: waist to height ratio

## References

[1] Haththotuwa RN, Wijeyaratne CN, Senarath U. Worldwide epidemic of obesity. In Obesity and obstetrics 2020; 3-8. Elsevier.

[2] Heidari-Beni M, Kelishadi R (2019). Prevalence of weight disorders in Iranian children and adolescents. Arch Iran Med..

[3] Sarokhani D, Sarokhani M, Dehkordi AH, Gheshlagh RG, Fakhri M (2020). Prevalence of obesity and overweight in Iranian students: a systematic review and meta-analysis. J Pediatr Endocrinol Metab..

[4] Yach D, Hawkes C, Gould CL, Hofman KJ (2004). The global burden of chronic diseases: overcoming impediments to prevention and control. JAMA..

[5] Krebs NF, Jacobson MS (2003). Prevention of pediatric overweight and obesity. Pediatrics..

[6] Dehghan M, Akhtar-Danesh N, Merchant AT (2005). Childhood obesity, prevalence and prevention. Nut J..

[7] Alexy U, Sichert-Hellert W, Kersting M, Schultze-Pawlitschko V (2004). Pattern of long-term fat intake and BMI during childhood and adolescence—results of the DONALD Study. Int J Obes Relat Metab Disord..

[8] Tucker LA, Kano MJ (1992). Dietary fat and body fat: a multivariate study of 205 adult females. Am J Clin Nutr..

[9] Atkin L-M, Davies PS (2000). Diet composition and body composition in preschool children. Am J Clin Nutr..

[10] Hooper L, Abdelhamid A, Bunn D, Brown T, Summerbell CD, Skeaff CM (2015). Effects of total fat intake on body weight. Cochrane Database Syst Rev..

[11] Elliott SA, Truby H, Lee A, Harper C, Abbott RA, Davies PS (2011). Associations of body mass index and waist circumference with: energy intake and percentage energy from macronutrients, in a cohort of Australian children. Nutr J..

[12] Payab M, Kelishadi R, Qorbani M, Motlagh ME, Ranjbar SH, Ardalan G (2015). Association of junk food consumption with high blood pressure and obesity in Iranian children and adolescents: the CASPIAN-IV Study. J Pediatr (Rio J)..

[13] Khoshhali M, Heidari-Beni M, Qorbani M, Motlagh ME, Ziaodini H, Heshmat R, et al. Tri-ponderal mass index and body mass index in prediction of pediatric metabolic syndrome: the CASPIAN-V study. Arch Endocrinol Metab. 2020;64(2):171-178. doi: 10.20945/2359-3997000000206. 10.20945/2359-3997000000206 PMC10118948 32236304

[14] Androutsos O, Grammatikaki E, Moschonis G, Roma-Giannikou E, Chrousos G, Manios Y (2012). Neck circumference: a useful screening tool of cardiovascular risk in children. Pediatr Obes..

[15] Kelishadi R, Heidari-Beni M, Qorbani M, Motamed-Gorji N, Motlagh ME, Ziaodini H, et al. Association between neck and wrist circumferences and cardiometabolic risk in children and adolescents: The CASPIAN-V study. Nutrition. 2017;43-44:32-38. doi: 10.1016/j.nut.2017.06.009. 10.1016/j.nut.2017.06.009 28935142

[16] Mameli C, Krakauer NY, Krakauer JC, Bosetti A, Ferrari CM, Moiana N (2018). The association between a body shape index and cardiovascular risk in overweight and obese children and adolescents. PLoS One..

[17] Payab M, Qorbani M, Shahbal N, Motlagh ME, Hasani-Ranjbar S, Zahedi H, et al. Association of anthropometric indices with metabolic phenotypes of obesity in children and adolescents: the CASPIAN-V study. Front Endocrinol (Lausanne). 2019;10:786. doi: 10.3389/fendo.2019.00786. 10.3389/fendo.2019.00786 PMC6902658 31849834

[18] Naude CE, Visser ME, Nguyen KA, Durao S, Schoonees A (2018). Effects of total fat intake on bodyweight in children. Cochrane Database Syst Rev..

[19] Scholz A, Navarrete-Muñoz EM, García-de-la-Hera M, Fernandez-Somoano A, Tardon A, Santa-Marina L (2019). Association between trans fatty acid intake and overweight including obesity in 4 to 5-year-old children from the INMA study. Pediatr Obes..

[20] Javardi MSM, Madani Z, Movahedi A, Karandish M, Abbasi B (2020). The correlation between dietary fat quality indices and lipid profile with Atherogenic index of plasma in obese and non-obese volunteers: a cross-sectional descriptive-analytic case-control study. Lipids Health Dis..

[21] Santos-Silva J, Bessa RJB, Santos-Silva F (2002). Effect of genotype, feeding system and slaughter weight on the quality of light lambs: II. Fatty acid composition of meat. Livestock Production Science..

[22] Ding EL, Sun Q, Campos H, Hu FB. Lipophilic index of fatty acid fluidity in erythrocyte and plasma and risk of coronary heart disease. Circulation. 2008;118:S-1089.

[23] Suara SB, Siassi F, Saaka M, Foroshani AR, Asadi S, Sotoudeh G (2020). Dietary fat quantity and quality in relation to general and abdominal obesity in women: a cross-sectional study from Ghana. Lipids Health Dis..

[24] Chen J, Liu H (2020). Nutritional indices for assessing fatty acids: A mini-review. Int J Mol Sci..

[25] Kelishadi R, Ardalan G, Qorbani M, Ataie-Jafari A, Bahreynian M, Taslimi M (2013). Methodology and early findings of the fourth survey of childhood and adolescence surveillance and prevention of adult non-communicable disease in Iran: The CASPIAN-IV study. Int J Prev Med..

[26] Kuczmarski RJ, Ogden CL, Guo SS, Grummer-Strawn LM, Flegal KM, Mei Z, Wei R, Curtin LR, Roche AF, Johnson CL. 2000 CDC Growth Charts for the United States: methods and development. Vital Health Stat 2002;11(246):1-190 12043359

[27] Nascimento VG, Bertoli CJ, Gallo PR, Abreu LCd, Leone C. Tri-Ponderal Mass Index: A Screening Tool for Risk of Central Fat Accumulation in Brazilian Preschool Children. Medicina (Kaunas). 2019;55(9):577. doi: 10.3390/medicina55090577. 10.3390/medicina55090577 PMC6780244 31500381

[28] Gomez-Marcos MA, Gomez-Sanchez L, Patino-Alonso MC, Recio-Rodriguez JI, Gomez-Sanchez M, Rigo F (2018). A body shape index and vascular structure and function in Spanish adults (MARK study): A cross-sectional study. Medicine (Baltimore)..

[29] Kelishadi R, Majdzadeh R, Motlagh M-E, Heshmat R, Aminaee T, Ardalan G (2012). Development and evaluation of a questionnaire for assessment of determinants of weight disorders among children and adolescents: the Caspian-IV study. Int J Prev Med..

[30] Shafiee G, Qorbani M, Heshmat R, Mohammadi F, Sheidaei A, Motlagh ME, Mahdavi-Gorabi A, Ardalan G, Ahadi Z, Kelishadi R (2019). Socioeconomic inequality in cardio-metabolic risk factors in a nationally representative sample of Iranian adolescents using an Oaxaca-Blinder decomposition method: the CASPIAN-III study. J Diabetes Metab Disord..

[31] Esfahani FH, Asghari G, Mirmiran P, Azizi F (2010). Reproducibility and relative validity of food group intake in a food frequency questionnaire developed for the Tehran Lipid and Glucose Study. J Epidemiol..

[32] USDA. United States Department of Agriculture (USDA) food Composition Databases. Available from: https://fdc.nal.usda.gov/

[33] Japanese Lipid Bank. Available from :http://lipidbank.jp/cgi-bin/ main.cgi?id=ALL.

[34] Te Morenga L, Montez JM (2017). Health effects of saturated and trans-fatty acid intake in children and adolescents: Systematic review and meta-analysis. PLoS One..

[35] Liu Q, Lichtenstein AH, Matthan NR, Howe CJ, Allison MA, Howard BV (2017). Higher lipophilic index indicates higher risk of coronary heart disease in postmenopausal women. Lipids..

[36] Soltani N, Farhangi MA, Nikniaz L, Mahmoudinezhad M (2020). Association between a novel dietary lipophilic index (LI) with metabolic phenotypes in a community-based study in Tabriz-Iran. BMC Endocr Disord..

[37] Wu H, Ding EL, Toledo ET, Campos H, Baylin A, Hu FB (2013). A novel fatty acid lipophilic index and risk of CHD in US men: the health professionals follow-up study. Br J Nutr..

[38] Emken E, Rohwedder W, Dutton H, Dejarlais W, Adlof R, Mackin J (1979). Incorporation of deuterium-labeledcis-andtrans-9-octadecenoic acids in humans: Plasma, erythrocyte, and platelet phospholipids. Lipids..

[39] Tram L, Krogh Venø S, Dahm CC, H Thomsen B, Berg Johansen M, Overvad K, et al. Adipose tissue lipophilic index and risk of ischemic stroke—a Danish case-cohort study. Nutrients. 2018;10(11):1570. doi: 10.3390/nu10111570. 10.3390/nu10111570 PMC6267621 30360550

[40] Shi J, Wang Z, Zhang W, Niu Y, Lin N, Li X (2021). Neck circumference as an independent predictor for NAFLD among postmenopausal women with normal body mass index. Nutr Metab (Lond)..

[41] Kurtoglu S, Hatipoglu N, Mazicioglu MM, Kondolot M (2012). Neck circumference as a novel parameter to determine metabolic risk factors in obese children. Eur J Clin Invest..

[42] Preis SR, Massaro JM, Hoffmann U, D'Agostino RB, Levy D, Robins SJ (2010). Neck circumference as a novel measure of cardiometabolic risk: the Framingham Heart study. J Clin Endocrinol Metab..

[43] Hotamisligil GS (2017). Inflammation, metaflammation and immunometabolic disorders. Nature..

[44] Adjepong M, Pickens CA, Jain R, Harris WS, Annan RA, Fenton JI (2018). Association of whole blood n-6 fatty acids with stunting in 2-to-6-year-old Northern Ghanaian children: A cross-sectional study. PLoS One..

[45] Barbarich B, Willows N, Wang L, Clandinin M (2006). Polyunsaturated fatty acids and anthropometric indices of children in rural China. Eur J Clin Nutr..

[46] Lagoutte-Renosi J, Allemand F, Ramseyer C, Rabani V, Davani S (2021). Influence of antiplatelet agents on the lipid composition of platelet plasma membrane: A lipidomics approach with ticagrelor and its active metabolite. Int J Mol Sci..

[47] Van Meer G, Voelker DR, Feigenson GW (2008). Membrane lipids: where they are and how they behave. Nat Rev Mol Cell Biol..

[48] Hulbert AJ, Turner N, Storlien L, Else P (2005). Dietary fats and membrane function: implications for metabolism and disease. Biol Rev Camb Philos Soc..

[49] Grundy SM (1999). Hypertriglyceridemia, insulin resistance, and the metabolic syndrome. Am J Cardiol..

[50] Tsuda K (2016). Association of resistin with impaired membrane fluidity of red blood cells in hypertensive and normotensive men: an electron paramagnetic resonance study. Heart Vessels..

[51] Moleres A, Ochoa MC, Rendo-Urteaga T, Martínez-González MA, San Julián MCA, Martínez JA (2012). Dietary fatty acid distribution modifies obesity risk linked to the rs9939609 polymorphism of the fat mass and obesity-associated gene in a Spanish case–control study of children. Br J Nutr..

[52] van Dijk SJ, Feskens EJ, Bos MB, Hoelen DW, Heijligenberg R, Bromhaar MG (2009). A saturated fatty acid–rich diet induces an obesity-linked proinflammatory gene expression profile in adipose tissue of subjects at risk of metabolic syndrome. Am J Clin Nutr..

[53] Clarke SD (2004). The multi-dimensional regulation of gene expression by fatty acids: polyunsaturated fats as nutrient sensors. Curr Opin Lipidol..

[54] Mumme K, Stonehouse W (2015). Effects of medium-chain triglycerides on weight loss and body composition: a meta-analysis of randomized controlled trials. J Acad Nutr Diet..

[55] Lee J-H (2013). Polyunsaturated fatty acids in children. Pediatr Gastroenterol Hepatol Nutr..

